# Using Machine Learning to Detect Factors That Affect Homocysteine in Healthy Elderly Taiwanese Men

**DOI:** 10.3390/biomedicines13081816

**Published:** 2025-07-24

**Authors:** Pei-Jhang Chiang, Chih-Wei Tsao, Yu-Cing Jhuo, Ta-Wei Chu, Dee Pei, Shi-Wen Kuo

**Affiliations:** 1 Division of Urology, Department of Surgery, Tri-Service General Hospital, National Defense Medical University, Taipei 114202, Taiwan; peijhang@gmail.com (P.-J.C.); weisurger@gmail.com (C.-W.T.); wavinglibra1012@gmail.com (Y.-C.J.); 2 In-Service Master Program in Artificial Intelligence in Medicine, College of Medicine, Taipei Medical University, Taipei 110301, Taiwan; 3 Department of Obstetrics and Gynecology, Tri-Service General Hospital, National Defense Medical University, Taipei 114202, Taiwan; taweichu@gmail.com; 4 MJ Health Research Foundation, Taipei 114066, Taiwan; 5 Division of Endocrinology and Metabolism, Department of Internal Medicine, Fu Jen Catholic University Hospital, College of Medicine, Fu Jen Catholic University, New Taipei 242062, Taiwan; peidee@gmail.com; 6 The Division of Endocrinology and Metabolism, Department of Internal Medicine, Taipei Tzu Chi Hospital, Buddhist Tzu Chi Medical Foundation, New Taipei 231016, Taiwan

**Keywords:** machine learning, homocysteine, elderly men, Taiwanese

## Abstract

**Background**: Homocysteine (Hcy) is a sulfur-containing amino acid crucial for various physiological processes, with elevated levels linked to cardiovascular and neurological adverse conditions. Various factors contribute to high Hcy, and past studies of impact factors relied on traditional statistical methods. Recently, machine learning (ML) techniques have greatly improved and are now widely applied in medical research. This study used four ML methods to identify key factors influencing Hcy in healthy elderly Taiwanese men, comparing their accuracy using multiple linear regression (MLR). The study seeks to improve Hcy prediction accuracy and provide insights into relevant impact factors. **Methods**: A total of 468 healthy elderly men were studied in terms of 33 parameters using four ML methods: random forest (RF), stochastic gradient boosting (SGB), eXtreme gradient boosting (XGBoost), and elastic net (EN). MLR served as a benchmark. Model performance was assessed using SMAPE, RAE, RRSE, and RMSE. **Results**: All ML methods demonstrated lower prediction errors than MLR, indicating higher accuracy. By averaging the importance scores from the four ML models, C-reactive protein (CRP) emerged as the leading impact factor for Hcy, followed by GPT, WBC, LDH, eGFR, and sport volume (SV). **Conclusions**: Machine learning methods outperformed MLR in predicting Hcy levels in healthy elderly Taiwanese men. CRP was identified as the most crucial factor, followed by GPT/ALT, WBC, LDH, and eGFR.

## 1. Introduction

Homocysteine (Hcy) is a sulfur-containing amino acid essential to various physiological processes and is increasingly recognized as a key biomarker for various health risks, especially in relation to cardiovascular and neurological conditions [1,2,3,4]. Hcy is generated during the metabolism of methionine, an essential amino acid, and its conversion into other compounds requires vitamins B6, B12, and folate (vitamin B9) [5]. A deficiency in these vitamins can lead to elevated homocysteine levels in the blood, a condition known as hyperhomocysteinemia [6]. Elevated Hcy can result from genetic factors, dietary deficiencies, and certain lifestyle choices, such as smoking and excessive alcohol consumption, genetic predispositions, and poor dietary intake [7]. Research has shown a strong link between high homocysteine levels and an increased risk of cardiovascular diseases (CVD) [8].

Beyond cardiovascular concerns, high Hcy levels are associated with cognitive decline, neurodegenerative diseases like Alzheimer’s and dementia, mood disorders, migraines, osteoporosis, and pregnancy complications. Although routine Hcy screening is not widely recommended for asymptomatic individuals due to its uncertain predictive value for heart disease, it can be valuable for certain high-risk populations [9]. Further research is needed to clarify how elevated homocysteine contributes to different diseases and to investigate possible therapeutic interventions.

Many biochemical determinates have been studied in terms of their relation to Hcy, such as Folate (B9), Vitamin B12, B6, and B2 [10]. Other features like renal clearance also contribute to Hcy [11].

However, relatively few studies have sought to evaluate the impact of demographic, biochemical, and lifestyle features on Hcy. Recent advances in machine learning (ML) techniques have led to their widespread use in predicting biomarkers. For example, applied to the phase 3 trial NCT02008227, the predictive Biomarker Modeling Framework uncovered a simple decision-tree biomarker (via model distillation) based on early trial data that would have led to a 15% better survival risk for the selected patients, compared to the original trial outcomes [12]. Similarly, ML methods have been applied in the prediction of large-artery atherosclerosis, osteosarcoma, precision medicine, and therapeutic target discovery [13,14,15,16,17,18,19]. Only two previous studies have sought to apply ML methods for the prediction of Hcy, one assessing the impact of mineral intake and the other one finding that Hcy is the key metabolite associated with long-term exposure to metformin [20,21].

The present study used four different ML methods to identify the key factors impacting Hcy in healthy elderly Taiwanese men, while assessing the accuracy of the various ML methods against multiple linear regression (MLR).

## 2. Materials and Methods

### 2.1. Participants and Study Design

Part of the following context was published by our group previously [22]. The data used in this research were derived from the Taiwan MJ Cohort, a long-term, prospective health examination study conducted by the MJ Health Screening Centers in Taiwan [23]. These extensive health assessments encompass over 100 key biological markers, such as anthropometric data, blood analyses, and imaging procedures. In addition, all participants completed a self-administered questionnaire covering details about their personal and family medical history, current health condition, lifestyle factors, physical activity, sleep patterns, and dietary behaviors [24]. Only individuals who provided written informed consent are included in the MJ Health Database. The study protocol received ethical approval from the Institutional Review Board of the Tri-Service General Hospital (IRB No. TSGHIRB C202305051). All or part of the data used in this research were authorized by and received from MJ Health Research Foundation (Authorization Code: MJHRF2024024A). An initial total of 1,556,410 individuals was subjected to the following inclusion criteria:

1. Men older than 65 years old: This study specifically focused on elderly populations, as age is a key factor influencing both homocysteine levels and age-related health changes. Given the known physiological differences in sex hormones and their influence on homocysteine metabolism, only male participants were included to eliminate sex as a potential confounding variable.
2. No current medication for metabolic syndrome: Metabolic syndrome is a complex condition associated with systemic metabolic dysregulation. Excluding participants with this syndrome helped isolate the role of homocysteine in otherwise healthy elderly men, reducing the influence of confounding metabolic abnormalities.
3. No significant medical diseases: Those with cancer or long-term use of medications for hyperglycemia, hypertension, or hyperlipidemia were excluded, as these conditions and their treatments can significantly alter metabolic pathways, including homocysteine levels, potentially introducing bias.
4. Data completion: Individuals with incomplete data for variables essential to modeling and analysis were excluded to ensure model reliability and avoid imputation bias.

**Figure 1:**
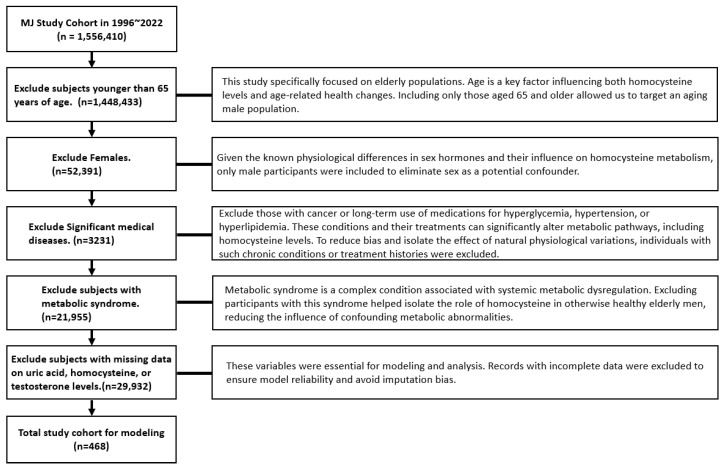
Participant selection process.

These exclusion criteria left a total of 468 participants for analysis (see Figure 1). Table 1 shows the independent variables (demographic, biochemical, and lifestyle information) and the dependent variable (Hcy) and their units. The methods used to gather demographic, biochemical, and lifestyle information were reported in our previous study and are not repeated here [22].

Instead of using smoking, sport, and drinking as categorical variables, the present study used the concept of area, where the variable “drinking area” is a derived measure representing cumulative alcohol exposure, constructed by multiplying four components: years of alcohol consumption, type or strength of alcohol consumed, amount typically consumed each time, and drinking frequency. This concept was similarly applied to smoking and sport activity.

Prior to analysis, the dataset was examined for the percentage of missing values across all relevant variables. No formal imputation was performed. Since our data were sourced from a dedicated health screening facility, most data records were complete. In the following statistical or ML methods, we performed the corresponding methods to adapt the missing data. For the standardization steps, all continuous variables (e.g., Hcy, BF, creatinine) were checked for normality. Skewed variables were log-transformed prior to analysis to meet model assumptions. Outliers were assessed using visual inspection (histograms, boxplots), and, where extreme values were likely due to measurement error, they were excluded based on pre-defined thresholds.

### 2.2. Traditional Statistics

Data are expressed as means ± standard deviations. To compare continuous variables between married and unmarried men, the Student’s *t*-test was applied. Pearson’s correlation analysis was used to evaluate the associations between Hcy levels and other continuous variables. For ordinal variables such as income and education levels, one-way analysis of variance (ANOVA) was performed. All statistical tests were two-tailed, and a *p*-value below 0.05 was considered indicative of statistical significance. Statistical analyses were carried out using SPSS version 10.0 for Windows (SPSS Inc., Chicago, IL, USA).

### 2.3. Proposed Machine Learning Scheme

This study introduces a predictive modeling approach for Hcy levels using four distinct machine learning (ML) techniques: random forest (RF), stochastic gradient boosting (SGB), eXtreme gradient boosting (XGBoost), and elastic net (EN). Please refer to our previous study for details [25].

RF is an ensemble learning algorithm based on decision trees, integrating bootstrap resampling with bagging techniques. Its core idea is to randomly generate multiple unpruned classification and regression trees (CART), using the reduction in Gini impurity as the criterion for node splitting. These individually trained trees are then aggregated to form a “forest.” The final prediction is made by averaging the outputs (for regression) or majority voting (for classification) across all trees, enhancing the model’s robustness and reducing overfitting.

Stochastic gradient boosting (SGB) is another tree-based ensemble method that combines aspects of bagging and boosting to minimize a defined loss function. Unlike RF, SGB builds trees sequentially, with each new tree trained to correct the residual errors made by the previous one. This process continues iteratively, using the residuals from each prior model as the input for the next, until a stopping criterion is met—such as a set number of iterations or convergence. The final model aggregates the outputs of all weak learners to produce a strong, stable predictive result.

XGBoost, the third algorithm used in this study, is an optimized and scalable implementation of gradient boosting. It improves upon traditional boosting methods by incorporating system and algorithmic enhancements. These include the use of second-order Taylor expansion to approximate the objective function, allowing for more efficient training with arbitrary differentiable loss functions. XGBoost also introduces regularization terms in its objective function to control model complexity, mitigate overfitting, and improve generalization accuracy.

EN, the final method used, is a regularized regression technique that combines both L1 (Lasso) and L2 (Ridge) penalties. This hybrid approach incorporates the sparsity-inducing property of Lasso, which can shrink some coefficients to zero for feature selection, with the grouping effect of Ridge, which stabilizes coefficient estimation when features are highly correlated. EN is especially effective when predictors are interrelated, as it tends to include grouped variables rather than arbitrarily selecting one. The main benefits of EN are the following: (1) it encourages grouped selection of correlated features, (2) it handles multicollinearity effectively, and (3) it balances feature selection and regularization to prevent overfitting while maintaining interpretability.

**Figure 2:**
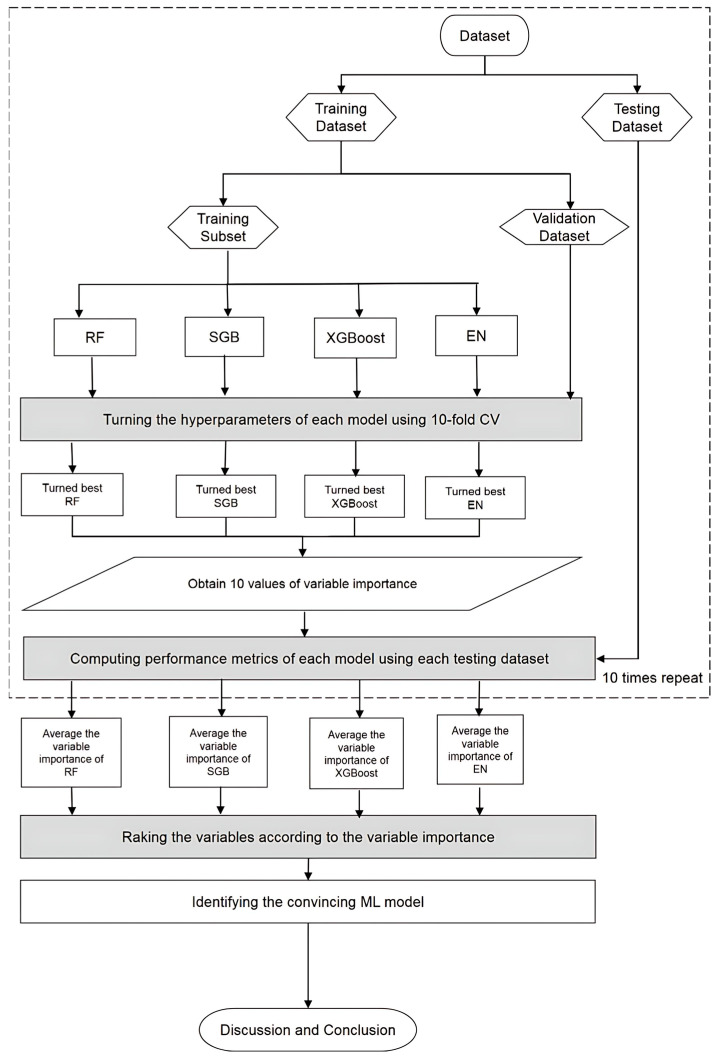
Proposed machine learning prediction scheme.

Figure 2 presents a flowchart of the proposed prediction framework and the key variable identification process that integrates all four machine learning methods. Initially, patient data were collected and preprocessed to construct the dataset. This dataset was then randomly split into 80% for training and 20% for testing. During model training, each machine learning algorithm underwent hyperparameter optimization through 10-fold cross-validation. A grid search was conducted across the parameter space to identify the best-performing configuration, with model selection based on the lowest root mean square error (RMSE) on the validation set. This established the most effective models for RF, SGB, XGBoost, and EN, and each model’s corresponding feature importance rankings were extracted.

Temporal hold-out validation was used to train the model for the results of validation. Since this is only a cross-sectional study, we simulated a temporal split. To evaluate the impacts and direction of the features, Shapley additive explanation was applied, and confidence intervals (CIs) of all methods were also calculated. To reveal systematic bias, a calibration plot was also performed. Finally, external validation was performed to assess the generalizability of a trained model.

Our results show that the root mean squared error (RMSE) ≈ 0.2 µmol/L. Reclassification analysis was performed to determine the distribution of participants.

During the testing phase, the performance of the optimized RF, SGB, XGBoost, and EN models was evaluated using the testing dataset. Since the target variable in this study is continuous, model performance was assessed using metrics such as symmetric mean absolute percentage error (SMAPE), relative absolute error (RAE), root relative squared error (RRSE), and root mean squared error (RMSE).

To ensure reliability and robustness of the models, the training and testing procedures were repeated 10 times with different random splits. The average performance metrics from these iterations were used to compare each model against a benchmark multiple linear regression (MLR) model, which was trained and tested using the same dataset splits. Any machine learning (ML) model that achieved lower average metric values than MLR was considered a convincing or superior model.

As each of the ML methods applied in this study is capable of generating a ranking of predictor variable importance, variables were ranked within each model from 1 (most important) to 30 (least important). However, due to the distinct mechanisms underlying each ML method, the variable importance rankings may differ across models. To enhance the reliability and consistency with which key risk factors were identified, the rankings from all convincing ML models were integrated.

In the final step of the proposed framework, significant insights were summarized and discussed based on the results of the convincing ML models, enabling the identification of key predictors contributing to homocysteine (Hcy) levels.

All analyses in this study were performed using R software version 4.0.5 and RStudio version 1.1.453. The ML models were implemented using the following R packages: “Random Forest” version 4.6-14 for RF [26], “gbm” version 2.1.8 for SGB [27], and “xgboost” version 1.5.0.2 for XGBoost [28]. Hyperparameter optimization for NB, RF, XGBoost, and EN models was carried out using the “caret” package version 6.0-90 [29]. The benchmark MLR model was constructed using the “stats” package version 4.0.5 with default settings.

### 2.4. Ethics Statement

The study protocol was approved by the institutional review board of Tri-Service General Hospital (IRB No. TSGHIRB C202305051). Informed consent was confirmed (or waived) by the IRB.

## 3. Results

A total of 468 subjects were enrolled, having first been screened for use of medication related to metabolic syndrome or significant medical disease (Figure 1).

The definitions and units of the study variables (independent) are presented in Table 1. Table 2 depicts the mean and standard deviation of these variables. Table 3 provides the Pearson correlation results, with age, platelet (Plt), alkaline phosphatase (Alp), lactic dehydrogenase (LDH), and uric acid (UA) all found to be positively correlated to Hcy level, while body fat (BF), serum glutamic pyruvic transaminase (SGPT/ALT), estimated glomerular filtration rate (eGFR), and LDL-cholesterol (LDL-C) were negatively correlated.

The average performance of the four ML methods and MLR is shown in Table 4. Each of the ML methods had smaller errors than MLR, indicating higher performance and greater accuracy. The confidence intervals (CI) of all methods are displayed in Table 5.

**Figure 3:**
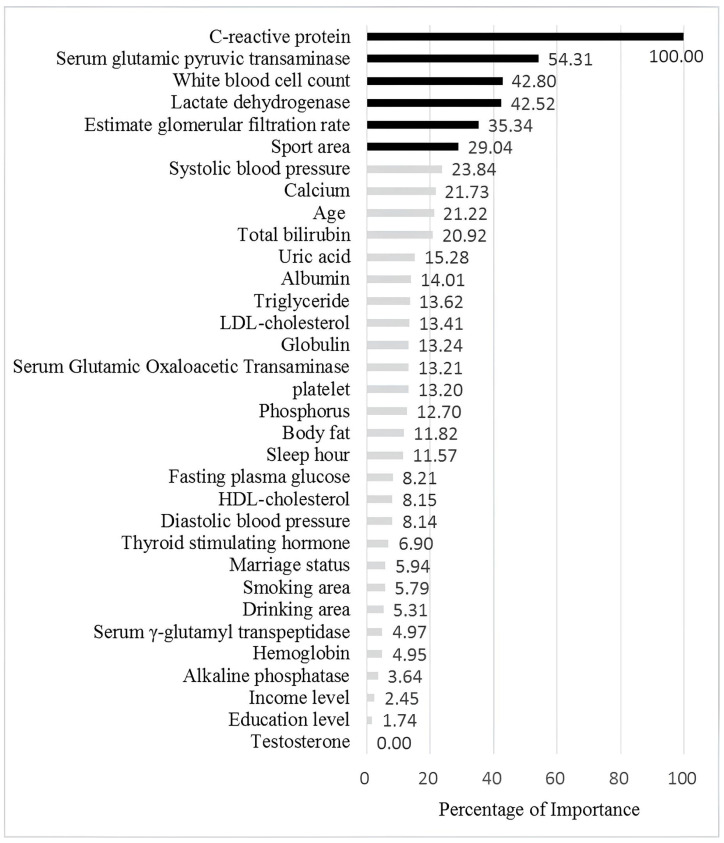
The figure illustration by relative importance of the different variables. The black bars indicate the most important six features for Hcy level, while the grey bars represent less important features.

Table 6 ranks the variables for each factor in terms of importance as determined by the RF, SGB, XGBoost, and EN models. As shown, the relative importance of variables varied across the different machine learning methods. The rightmost column of the table presents the average importance scores for each variable, providing a consolidated view. Figure 3 offers a graphical representation of these results, with variables arranged from top to bottom in order of descending importance. As shown in Figure 3, C-reactive protein (CRP) ranked among the top contributors, followed by GPT, WBC, LDH, eGFR, and sport area in healthy elderly Taiwanese men.

**Figure 4:**
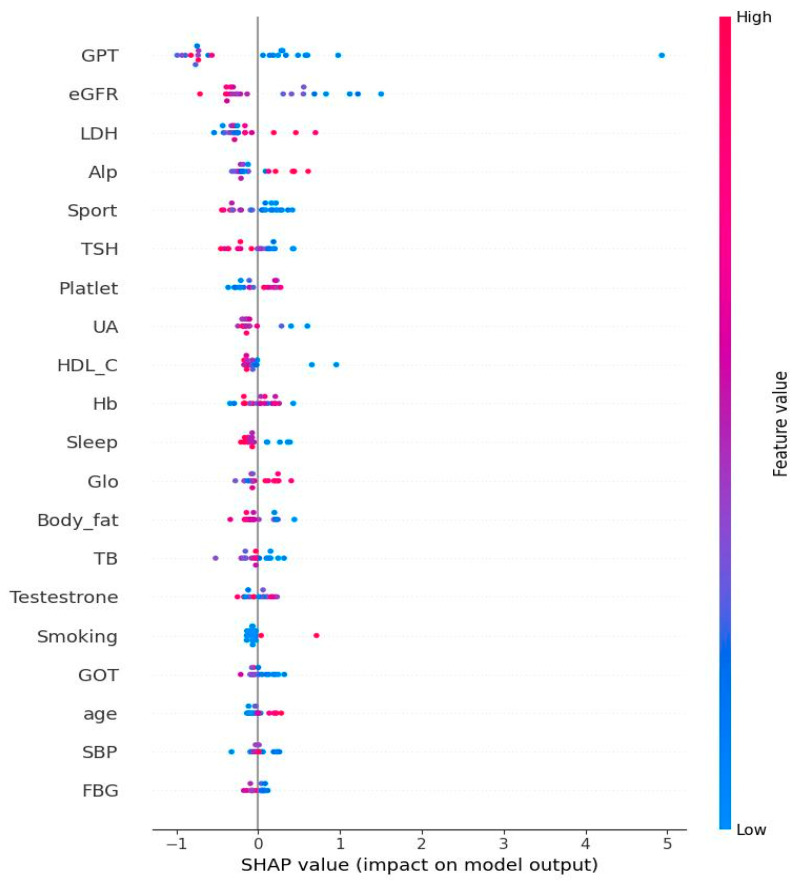
Bee swarm from Shapley additive explanation of stochastic gradient boosting. Note: The features at the top of the figure are the most important ones. The use of red in the diagram indicates a high impact on the homocysteine level. SGPT/ALT: serum glutamic pyruvic transaminase; eGFR: estimated glomerular filtration rate; LDH: lactate dehydrogenase; ALP: alkaline phosphatase; TSH: thyroid-stimulating hormone; UA: uric acid; HDL-C: high-density lipoprotein cholesterol; Hb: hemoglobin; Glo: globulin; TBIL: total bilirubin; plasma glucose; TBIL: total bilirubin; SGOT/AST: serum glutamic oxaloacetic transaminase; SBP: systolic blood pressure; FPG: fasting plasma glucose.

Figure 4 shows the Bee swamp plot derived from SHAP of SBG. Each ML method has its own SHAP plot. We chose SBG since it has the lowest average performance compared to other methods. The important features are consistent with the results shown in Table 3. The discrepancy is from that each model has its own algorithm for calculation. In Figure 4, the Bee swamp plot derived from SHAP of SBG is displayed. It should be noted that each ML method has its own SHAP plot. We chose SBG since it has the least average performance compared to other methods. From this figure, it could be noted that the important features are very similar to Table 3. The discrepancy is from the different algorithms in SGB and SHAP.

The present study is cross-sectional. Thus, to perform temporal hold-out testing, we split the data by sorting the data of enrolling the participants, with results shown in Table 7.

**Figure 5:**
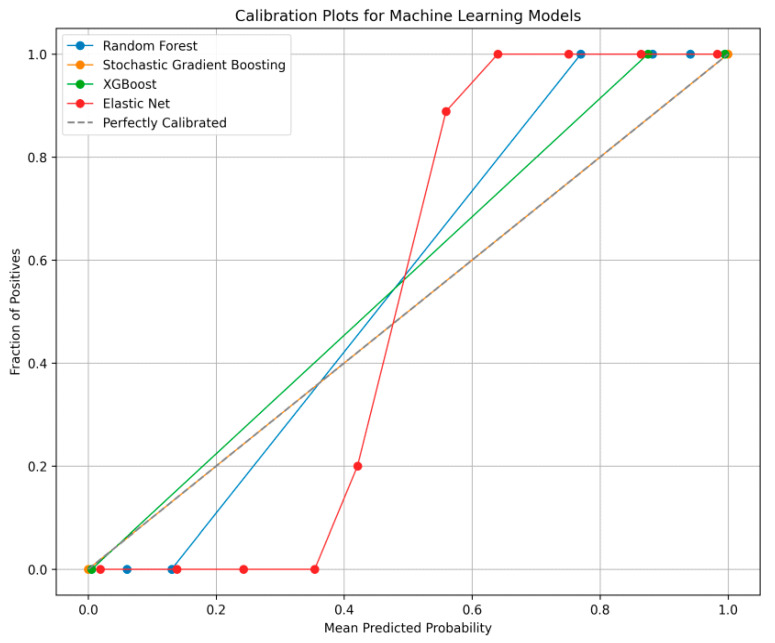
Calibration plot for machine learning methods.

To determine how well the predicted probabilities of the different machine learning models correspond to actual outcomes, Figure 5 presents a calibration plot. SGB provides the best performance in terms of alignment with the dashed diagonal line, while EN performs the poorest.

**Figure 6:**
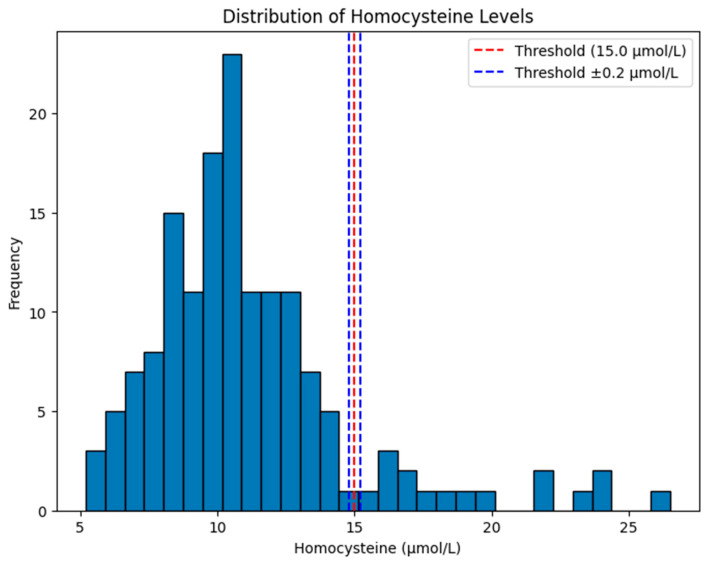
Distribution of homocysteine level in our study cohort under root-mean-squared error ≈ 0.2 µmol/L.

Finally, in Figure 6, the histogram shows the distribution of homocysteine levels in our dataset, with emphasis on a clinical threshold value of 15.0 µmol/L and a margin of ±0.2 µmol/L. The red dashed line is the threshold at 15.0 µmol/L, a cutoff commonly used to define hyperhomocysteinemia. The blue dashed lines have margins at 14.8 µmol/L and 15.2 µmol/L, showing a small range around the threshold (±0.2 µmol/L) that could be used for sensitivity analysis or as a classification boundary.

## 4. Discussion

Few previous studies have used machine learning techniques to assess potential risk factors for elevated Hcy levels, and those focused on the impact of mineral intake or metformin exposure, which are not relevant to the goals of our present study. Our results identify key predictors for Hcy levels in a cohort of healthy elderly men not currently using medications or suffering from significant medical conditions that could affect the independent variables used in the study.

The present study uses a cross-sectional design, and thus no causal relationships can be determined. However, multiple studies have demonstrated that elevated Hcy levels stimulate pro-inflammatory signaling in vascular and other cell types. For example, Hcy promotes the expression of adhesion molecules, chemokines, and cytokines via redox-sensitive mechanisms, leading to leukocyte recruitment and vascular inflammation. This indicates that inflammation is not just a downstream effect but is actively triggered by Hcy [30,31].

Table 4 and Table 5 show the performance metrics and CI for our methods. The performance metric is just a point estimate and does not indicate the degree of uncertainty or variation that might result from repeating the study. At the same time, CI shows the reliability and robustness of each model.

In the present study, the simple correlation results do not exactly match the final ML results. In other words, some of the related factors were not selected by ML methods. Despite this, we argue that the ML results are more accurate since they capture the non-linear relationships between variables and the models were adjusted for the internal impacts among these variables.

While CRP did not appear to have a significant effect in the simple correlation, the present study finds it is one of the most important factors related to Hcy. CRP is well known as an important biomarker for inflammation and cardiovascular disease [32], similar to Hcy [33]. In an animal study, Pang et al. showed that Hcy can initiate an inflammatory response in vascular smooth muscle cells by stimulating CRP production via the N-methyl-D-aspartate receptor (NMDAr) and subsequent generation of reactive oxygen species. In a review article, Ganguly et al. also reported that Hcy is directly related to both nervous and cardiovascular diseases [1]. The results of the present study are in line with these findings and support this relationship in healthy elderly Taiwanese men.

GPT is an important enzyme mainly found in the liver. It is crucial for amino acid metabolism and is a biomarker for hepatitis [34]. The liver also plays an important role in the metabolism of Hcy, converting it into methionine and cysteine. Abnormal liver function (including hepatitis) would result in increased Hcy levels [35]. Among the variables examined in the present study, GPT is found to be the second most significant impact factor for Hcy level, further consolidating this relationship. GPT is found to be a more significant impact factor than GOT, and this could be explained by their relative distributions, as GPT is more liver-specific, while GOT is also found in the heart, kidneys, and muscles [36]. Thus, it could be concluded that GPT is more useful than GOT as a specific marker for liver disease.

Hcy is widely regarded as an inflammatory marker in humans. However, only a few studies have found a positive correlation between Hcy and WBC, particularly Carru et al., but their study only used a cohort of 124 participants. The present study, using a considerably larger cohort, found the same result. This relationship could be explained by the influence of homocysteine on WBC counts, which affect the endothelial function and vascular health [37,38]. Our finding further explains the role played by Hyc in inflammation and chronic disease management.

LDH was found to be the 4th most important factor for Hcy levels. In an animal study, Samra et al. successfully explained this relationship by showing that Hcy activates glycolytic enzymes, including LDH, in retinal pigment epithelial cells [39]. Reversely, treating hyper-homocysteinemia could improve the aforementioned conditions even under stress conditions. The present study further consolidates this relationship, and further longitudinal study would be needed to demonstrate the causal relationship.

Inconsistent with previous studies, our results show a negative correlation between Hcy and eGFR. In a review article, Guldener suggested this was due to impaired renal clearance for Hyc [11]. In addition, Hcy could induce oxidative stress [40] and trigger endoplasmic reticulum stress in renal cells [41]. Our contrary finding could be due to the following facts:

1. Hcy levels increase above the age of 50, and the subjects of the present study are all elderly men with higher Hcy levels, while their eGFR levels declined with age.
2. Men generally have higher homocysteine levels than women in all age groups [42].
3. Hcy levels vary across ethnic groups, with Cappucio et al. reporting that South Asians had significantly higher Hcy levels as compared to Caucasians [43].

The finding of the present study provides another aspect of the relationship between Hcy and eGFR in the elderly.

Lastly, we found that sport area (exercise intensity X years of exercise X weekly hours of exercise) was the least important factor for Hcy, showing a negative but not insignificant relationship in simple correlation. While the findings of Tsai et al. supported those reported here, this relationship remains controversial [44], but discrepancies may be due to differences in the ethnic characteristics of study participants, the methods of analysis, and various exercise types (acute, chronic, or resistant) [45]. Finally, it should be noted that the present study’s notion of ‘sport area’ provides a more accurate means of quantification than those used in most other studies.

The present study is subject to certain limitations. First, in terms of potential selection bias, participants with missing values for key variables (e.g., homocysteine or covariates) were excluded, which may have resulted in a healthier, more compliant subset of the original sample, and individuals who participate in research often differ from the general population in terms of health behaviors, socioeconomic status, and disease burden, potentially underestimating associations with Hcy. Second, while the study does not include data for folate, vitamin B6/B12, and MTHFR genotype, the primary aim of the analysis was to investigate the relationship between Hcy and modifiable demographic, lifestyle, and biochemical factors, and meaningful associations can still be identified and interpreted, even in the absence of these biomarkers. Third, the cross-sectional nature of our study limits its use in making causal or temporal inferences between the predictors and homocysteine levels. As such, we cannot determine whether the identified features are causes or consequences of elevated Hcy. However, the primary aim of this study was not to establish causality but rather to identify potential associations and important predictors of Hcy levels using ML approaches. Our findings can be used to generate hypotheses for future longitudinal or interventional studies that are better suited for establishing temporality and causality.

## 5. Conclusions

Machine learning (ML) methods were shown to outperform traditional MLR in identifying association of risk factors for elevated Hcy levels in healthy, elderly Taiwanese men. The most important risk factor is identified as CRP, followed by GPT, WBC, LDH, eGFR, and sport area.

## Figures and Tables

**Table 1:** Variable units and description.

Variables	Unit and Description
Age	Years
Marital status (MS)	(1) Unmarried (2) Married
Income level (IL)	NTD/year (1) Below USD 200,000 (2) USD 200,001–USD 400,000 (3) USD 400,001–USD 800,000 (4) USD 800,001–USD 1,200,000 (5) USD 1,200,001–USD 1,600,000 (6) USD 1,600,001–USD 2,000,000 (7) More than USD 2,000,000
Education level (Edu.)	(1) Illiterate; (2) Elementary school; (3) Junior high school; (4) High school (vocational); (5) Junior college; (6) University; (7) Graduate school or above
Body fat (BF)	%
Systolic blood pressure (SBP)	mmHg
Diastolic blood pressure (DBP)	mmHg
Leukocyte (WBC)	×10^3^/μL
Hemoglobin (Hb)	×10^6^/μL
Platelets (Plt)	×10^3^/μL
Fasting plasma glucose (FPG)	mg/dL
Total bilirubin (TBIL)	mg/dL
Albumin (Alb)	mg/dL
Globulin (Glo)	mg/dL
Alkaline phosphatase (ALP)	IU/L
Serum glutamic oxaloacetic transaminase (SGOT/AST)	IU/L
Serum glutamic pyruvic transaminase (SGPT/ALT)	IU/L
Serum γ-glutamyl transpeptidase (γ-GT)	IU/L
Lactate dehydrogenase (LDH)	IU/L
Estimated glomerular filtration rate (eGFR)	mg/dL
Uric acid (UA)	mg/dL
Triglycerides (TG)	mg/dL
High-density lipoprotein cholesterol (HDL-C)	mg/dL
Low-density lipoprotein cholesterol (LDL-C)	mg/dL
Calcium (Ca)	mg/dL
Phosphorus (P)	mg/dL
Thyroid-stimulating hormone (TSH)	IU/mL
C-reactive protein (CRP)	mg/dL
Testosterone (T)	ng/ml
Drinking area	-
Smoking area	-
Sport area	-
Sleeping hours (SH)	(1) 0~4 h (2) 4~6 h (3) 6~7 h (4) 7~8 h (5) 8~9 h (6) more than 9 h
Homocysteine (Hcy)	μmol/L

**Table 2:** The mean and standard variations of the demographic, biochemical, and lifestyle variables.

Numeric Variable	Mean ± SD	Ordinal Variables	N (%)
Age	69.69 ± 4.68	Marital status (MS)	
Body fat (BF)	21.52 ± 5.79	(1) Unmarried	68 (16.39%)
Systolic blood pressure (SBP)	129.35 ± 19.22	(2) Married	347 (83.61%)
Diastolic blood pressure (DBP)	78.92 ± 10.95	Income level (IL)	
Leukocyte (WBC)	5.60 ± 1.39	(1) Below USD 200,000	51 (28.18%)
Hemoglobin (Hb)	14.82 ± 1.24	(2) USD 200,001–USD 400,000	29 (16.02%)
Platelets (Plt)	197.95 ± 50.82	(3) USD 400,001–USD 800,000	44 (24.31%)
Fasting plasma glucose (FPG)	109.47 ± 21.38	(4) USD 800,001–USD 1,200,000	32 (17.68%)
Total bilirubin (TBIL)	1.16 ± 0.41	(5) USD 1,200,001–USD 1,600,000	104 (5.52%)
Albumin (Alb)	4.30 ± 0.21	(6) USD 1,600,001–USD 2,000,000	4 (2.21%)
Globulin (Glo)	3.08 ± 0.35	(7) More than USD 2,000,000	11 (6.08%)
Alkaline Phosphatase (ALP)	62.24 ± 16.72	Education level (Edu.)	
Serum glutamic oxaloacetic transaminase (SGOT/AST)	25.61 ± 9.01	(1) Illiterate	8 (1.94%)
Serum glutamic pyruvic transaminase (SGPT/ALT)	26.49 ± 15.57	(2) Elementary school	95 (23.00%)
Serum γ-glutamyl transpeptidase (γ-GT)	30.96 ± 31.42	(3) Junior high school	51 (12.35%)
Lactate dehydrogenase (LDH)	171.91 ± 29.59	(4) High school (vocational)	82 (19.85%)
Estimated glomerular filtration rate (eGFR)	72.43 ± 12.09	(5) Junior college	62 (15.01%)
Uric acid (UA)	6.16 ± 1.28	(6) University	78 (18.89%)
Triglycerides (TG)	109.07 ± 54.75	(7) Graduate school or above	37 (8.96%)
High-density lipoprotein cholesterol (HDL-C)	54.47 ± 12.80	Sleeping hours (SH)	
Low-density lipoprotein cholesterol (LDL-C)	121.14 ± 32.80	(1) 0~4 h	24 (5.45%)
Calcium (Ca)	9.40 ± 0.40	(2) 4~6 h	120 (27.27%)
Phosphorus (P)	3.37 ± 0.42	(3) 6~7 h	182 (41.36%)
Thyroid-stimulating hormone (TSH)	1.83 ± 1.22	(4) 7~8 h	83 (18.86%)
C-reactive protein (CRP)	0.23 ± 0.40	(5) 8~9 h	26 (5.91%)
Testosterone (T)	5.85 ± 2.34	(6) more than 9 h	5 (1.14%)
			
		Dependent variable	Mean ± SD
		Homocysteine (Hcy)	11.05 ± 3.81

**Table 3:** Simple correlation between homocysteine and other independent variables.

	Age	BF	SBP	DBP	WBC	Hb	Plt
Hcy	0.181 ***	−0.104 *	0.123 **	0.126 **	0.128 **	−0.077	0.137 **
	FPG	TBIL	Alb	Glo	ALP	SGOT/AST	SGPT/ALT
Hcy	−0.025	−0.071	0.023	0.060	0.108 *	−0.064	−0.134 **
	γ-GT	LDH	eGFR	UA	TG	HDL-C	LDL-C
Hcy	−0.028	0.222 ***	−0.258 ***	0.091 *	0.051	−0.048	−0.151 **
	Ca	P	TSH	CRP	T	Drink area	
Hcy	0.077	0.059	−0.061	0.056	0.009	0.001	
	Smoke area			Sport area		SH	
Hcy	0.127 **			−0.024		−0.021	

Hcy: homocysteine; BF: body fat; SBP: systolic blood pressure; DBP: diastolic blood pressure; WBC: leukocyte; Hb: hemoglobin; Plt: platelets; FPG: fasting plasma glucose; TBIL: total bilirubin; Alb: albumin; Glo: globulin; ALP: alkaline phosphatase; SGOT/AST: serum glutamic oxaloacetic transaminase; SGPT/ALT: serum glutamic pyruvic transaminase; γ-GT: serum γ-glutamyl transpeptidase; lactate dehydrogenase (LDH); eGFR: estimated glomerular filtration rate; UA: uric acid; TG: triglycerides; HDL-C: high-density lipoprotein cholesterol; LDL-C: low-density lipoprotein cholesterol; Ca: calcium; P: phosphorus; TSH: thyroid-stimulating hormone; CRP: C-reactive protein; T: testosterone; SH: sleeping hours. * *p* <0.05; ** *p* < 0.005, *** *p* < 0.001

**Table 4:** Average performance of the four machine learning methods and multiple linear regression methods.

Methods	SMAPE	RAE	RRSE	RMSE
MLR	0.3476 [0.3458–0.3512]	0.3621 [0.3578–0.3641]	1.1483 [1.058–1.2014]	1.1856 [1.1296–1.2403]
RF	0.2863 [0.2780–0.3021]	0.2751 [0.2701–0.2865]	0.9601 [0.9510–0.9724]	0.9778 [0.9601–0.9825]
SGB	0.2656 [0.2558–0.2857]	0.2602 [0.2564–0.2714]	0.9106 [0.9001–0.9210]	0.9557 [0.9420–0.9618]
XGBoost	0.2765 [0.2650–0.2814]	0.2629 [0.2520–0.2769]	0.9315 [0.9211–0.9468]	0.9699 [0.9510–0.9752]
EN	0.2566 [0.2451–0.2687]	0.2557 [0.2414–0.2667]	0.8901 [0.8810–0.9015]	0.9652 [0.9541–0.9762]

Data showed as mean values; MLR: multiple linear regression; RF: random forest; SGB: stochastic gradient boosting; XGBoost: eXtreme gradient boosting; EN: elastic net; SMAPE: symmetric mean absolute percentage error; RAE: relative absolute error; RRSE: root relative quared error; RMSE: root mean squared error.

**Table 5:** Confidence interval of the methods used in the present study derived from Shapley addictive explanation.

	MAPE_CI	SMAPE_CI	RAE_CI	RRSE_CI	RMSE_CI	R^2^_CI
MLR	[0.2369, 0.8151]	[0.2428, 0.4525]	[0.8052, 1.3704]	[0.8059, 1.2958]	[1.7084, 2.8818]	[−0.6791, 0.3505]
EN	[0.2288, 0.7930]	[0.2304, 0.4394]	[0.7829, 1.3143]	[0.7799, 1.2569]	[1.6599, 2.7939]	[−0.5797, 0.3918]
RF	[0.1985, 0.8344]	[0.1867, 0.3956]	[0.7735, 1.1618]	[0.8900, 1.1780]	[1.6326, 2.9791]	[−0.3876, 0.2080]
SGB	[0.2028, 0.7324]	[0.1936, 0.4015]	[0.7240, 1.2011]	[0.8517, 1.2039]	[1.6484, 3.0692]	[−0.4495, 0.2746]
XGBoost	[0.2219, 0.7133]	[0.1991, 0.4055]	[0.7612, 1.2891]	[0.8507, 1.3298]	[1.7680, 2.9339]	[−0.7683, 0.2762]

Data showed as means; MLR: multiple linear regression; EN: elastic net; RF: random forest; SGB: stochastic gradient boosting; XGBoost: eXtreme gradient boosting; MAPE: mean absolute percentage error; SMAPE: symmetric mean absolute percentage error; RAE: relative absolute error; RRSE: root relative squared error; RMSE: root mean squared error.

**Table 6:** Relative importance from four different machine learning methods and their average.

	RF	SGB	XGBoost	EN	Average
Age	25.63	30.92	16.04	12.29	21.22
Marital status	1.92	8.72	1.98	11.12	5.94
Income level	6.95	0.00	2.84	0.00	2.45
Education level	4.46	0.00	2.48	0.00	1.74
Body fat	10.93	0.00	36.33	0.00	11.82
Systolic blood pressure	53.03	7.24	32.43	2.66	23.84
Diastolic blood pressure	20.26	8.11	4.19	0.00	8.14
White blood cell count	49.52	16.93	12.90	91.86	42.80
Hemoglobin	10.30	0.00	9.49	0.00	4.95
platelet	18.43	22.66	11.72	0.00	13.20
Fasting plasma glucose	14.74	11.57	6.51	0.00	8.21
Total bilirubin	34.27	10.22	13.01	26.19	20.92
Albumin	5.12	0.00	0.99	49.93	14.01
Globulin	15.97	11.46	25.52	0.00	13.24
Alkaline phosphatase	12.46	0.00	2.09	0.00	3.64
Serum Glutamic Oxaloacetic Transaminase	25.79	5.37	21.67	0.00	13.21
Serum glutamic pyruvic transaminase	75.28	56.59	77.32	8.06	54.31
Serum γ-glutamyl transpeptidase	14.26	0.00	5.61	0.00	4.97
Lactate dehydrogenase	72.35	31.92	65.20	0.59	42.52
Estimate glomerular filtration rate	55.68	29.83	44.20	11.65	35.34
Uric acid	17.30	26.84	16.98	0.00	15.28
Triglyceride	16.64	20.41	17.44	0.00	13.62
HDL-cholesterol	15.74	8.65	7.92	0.27	8.15
LDL-cholesterol	24.40	9.88	19.37	0.00	13.41
Calcium	13.10	0.00	13.33	60.50	21.73
Phosphorus	18.31	0.00	32.47	0.00	12.70
Thyroid-stimulating hormone	13.10	4.44	10.07	0.00	6.90
C-reactive protein	100.00	100.00	100.00	100.00	100.00
Testosterone	0.00	0.00	0.00	0.00	0.00
Drinking area	3.21	17.91	0.12	0.00	5.31
Smoking area	8.87	0.00	11.69	2.61	5.79
Sport area	9.22	20.81	12.93	73.21	29.04
Sleep hour	16.71	11.86	17.71	0.00	11.57

RF: random forest; SGB: stochastic gradient boosting; XGBoost: eXtreme gradient boosting; EN: elastic net.

**Table 7:** Temporal hold-out validation test results.

	RF	SGB	XGBoost	EN
RMSE	3.7219	3.7956	3.9459	3.8626
R^2^	0.1156	0.0802	0.006	0.0475

Data are shown as means; RF: random forest; SGB: stochastic gradient boosting; XGBoost: eXtreme gradient boosting; EN: elastic net; RMSE: root mean squared error.

## Data Availability

Data available on request due to privacy/ethical restrictions. This study used secondary databases for analysis, sourced from the MJ Health Research Foundation.
